# Role of Intracellular Adhesion icaAD and agr genes in Biofilm Formation in Clinical S. aureus Isolates and Assessment of Two Phenotypic Methods

**DOI:** 10.12669/pjms.343.14530

**Published:** 2018

**Authors:** Tayyeba Saba, Muhammad Sajid, Amir Afzal Khan, Rabaab Zahra

**Affiliations:** 1 Tayyeba Saba, M.Phil, Department of Microbiology, Quaid-i-Azam University, Islamabad, Pakistan; 2 Muhammad Sajid, PhD. School of Mechanical and Manufacturing Engineering (SMME), National University of Sciences and Technology (NUST), Islamabad, Pakistan; 3 Amir Afzal Khan, M.Phil. Department of Microbiology, Quaid-i-Azam University, Islamabad, Pakistan; 4 Rabaab Zahra, PhD. Department of Microbiology, Quaid-i-Azam University, Islamabad, Pakistan

**Keywords:** *agr*, Biofilm, *S. aures*, Simulations

## Abstract

**Objective:**

To determine the role of *icaAD* and *agr* genes in biofilm formation and evaluate the consistency of two phenotypic methods for biofilm measurement.

**Methods:**

A total of 81 clinical *S. aureus* strains were included and analyzed for biofilm formation by two methods. The microtitration plate method was optimized using computational fluid dynamics and compared with the Congo red assay. The genes for *icaAD* and *agr* were detected using PCR.

**Results:**

Of 81 isolates, biofilm production was detected in 43% isolates using Congo red method while microtiter plate assay showed biofilm production in 92% isolates. Both methods showed correlation in 30% isolates. PCR detection showed *icaAD* gene in 42 (52%) isolates. Out of 81 *S. aureus* isolates 65 strains (80%) contained *agr* while 16 (20%) strains were non-typeable.

**Conclusions:**

In conclusion, biofilm production was observed for both *agr* positive and *agr* negative isolates. Furthermore, the presence of *icaAD* genes was not associated with all biofilm producing strains as some strains negative for *icaAD* genes displayed biofilm production.

## INTRODUCTION

*Staphylococcus aureus* (*S. aureus*) is an important human pathogen which causes both hospital and community acquired infections and under circumstances can be opportunistic pathogen. Severity of infections by *S. aureus* ranges from localized skin abscesses to life threatening diseases like osteomyelitis, arthritis, endocarditis and infections associated with colonization of medical devices like intravenous catheters and heart valves.^1^

Major reason for biomaterial associated infections is the formation of biofilm in which these organisms adhere to medical devices.^2^Biofilm formation takes place in two steps: first microorganism adhere to substrate surface and then cells stick to each other.^3^ *Ica* operon in *S. aureus* contains *icaADBC* genes which play an important role in biofilm formation.^4^ N-acetylglucosaminyltransferase enzyme is encoded by *icaA* gene and is responsible for polysaccharide intracellular adhesion (PIA) production, while *icaD* is involved in enhancing the activity of the enzyme for complete phenotypic expression of PIA.^5^ A number of infections are caused by biofilm forming *S. aureus* so it is important to have reliable method for the early detection of biofilm forming strains.^6^

Microtiter plate assay is considered as standard screening method for comparing adherence pattern and is most widely used quantitative method for detection of biofilm formation.^7^,^8^ Computational fluid dynamics (CFD) simulations have been used to optimize the suitable conditions to detect the biofilm formation by this method and have proven to be efficient.^9^,^10^

In *S. aureus* accessory gene regulator (*agr*) locus encodes the quorum sensing systemwhich plays an important role in biofilm formation and has been shown to slow down biofilm formation.^11^,^12^ *S. aureus* strains having no *agr* group can form strong biofilm.^12^,^13^ When biofilm has formed activation of *agr* system is required for detachment of biofilm.

In view of importance of infections associated with biofilm formation, the aim of this study was to determine biofilm producing ability of clinical *Staphylococcus aureus* and to evaluate reliability of phenotypic methods.

## METHODS

### Bacterial isolates

A total of 81 *S. aureus* strains collected from Pakistan Institute of Medical Science (PIMS), Islamabad were included in the study. All isolates were confirmed by Gram staining, growth on mannitol salt agar and biochemical tests such as catalase, coagulase and DNase tests. Susceptibility testing to cefoxitin was conducted by disk diffusion method following the guidelines of Clinical and Laboratory Standards Institute (CLSI) 2016.

### Biofilm formation by Congo red agar (CRA) method

Congo red agar medium was prepared with 37 g/l of brain heart infusion broth (BHI), 10 g/l agar, 50 g/l sucrose and 0.8 g/l Congo red. Congo red stain solution was prepared and autoclaved separately from other medium constituents. Plates were inoculated with test organisms and incubated for 24 to 48hrs at 37ºC. PIA positive strains produced black colonies while PIA negative strains produced red colonies.

### Computational Fluid Dynamics (CFD) Simulations

**Fig.1:**
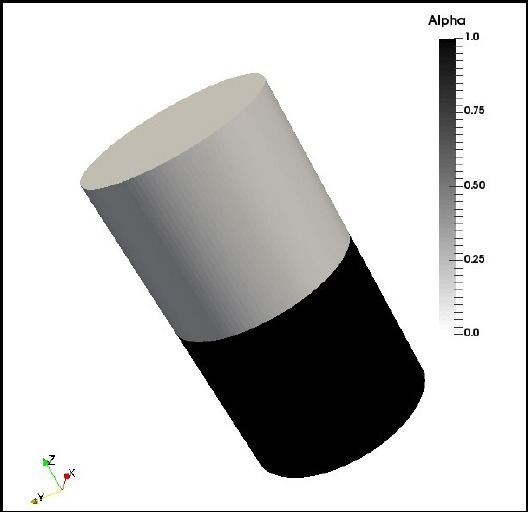
Phase distribution in the discretized domain used in the numerical study. Alpha: 1 corresponds with water.

The computational domain (6.6mm diameter and 11.6mm high cylindrical region that is filled with 200 µL of water) was modeled using the ‘blockMesh’ utility of the open source CFD Tool ‘OpenFOAM’^14^ leading to a structured mesh of 25,000 hexahedra elements, with maximum aspect ratio, non-orthogonality and skewness of 3.49282, 31.909 and 2.00744 respectively (Fig.1). A finite volume method (FVM) discretization was coupled to a volume-of-fluid (VoF) approach to describe the air-water two phase flows using the parameter α to determine the fraction of liquid in each mesh element. The revolving motion of the orbital shaker was implemented using an accelerating reference frame by introducing a source term in the momentum equation to capture the effects of the well motion.

### Biofilm formation by Microtiter plate (MTP) assay

Biofilm formation by *S. aureus* was determined using 96 well microtiter plate assays. Each strain was tested in triplicate. Plates were incubated for 48 hrs at 37C in shaking incubator at 130 rpm. Then wells were stained with 0.1% crystal violet solution for 10 minutes. In order to remove adherent bacteria from the wells, 200μl of 30% acetic acid was added to each well. The contents of the wells were mixed and optical density of each well was measured at 590 nm which indicated biofilm production. For interpretation of results, the strains were divided into three categories; moderate biofilm producers, strong biofilm producers and weak/no biofilm producers.^4^

### PCR Amplification and Detection of agr Groups and icaAD genes

For *agr* genes amplification, primers sequences were chosen from published sequences^15^ and PCR was performed as described previously.^16^ For *icaAD* genes PCR, the sequences and PCR conditions used were as already described.^17^ Amplified samples were analyzed by electrophoresis on a 1% agarose gel and stained with ethidium bromide.

## RESULTS

### Bacterial Isolates

A total of 81 clinical isolates were confirmed as *S. aureus* by using Gram staining and standard biochemical tests. All isolates were mannitol fermenters and positive for catalase, DNase and coagulase enzymes. Resistance to cefoxitin presented the prevalence of methicillin resistant *S. aureus* (MRSA) to be 55%.

### CFD Simulations

The numerical study of the micro-well indicated that the slope of the air/water interface stabilized after initial period of sloshing. Greater slopes were observed at higher orbital frequencies, which were also characterized by recirculation zones near the interface leading to greater convective mixing. This hydrodynamic mixing is responsible for transporting nutrients to growing biofilm and therefore our simulations suggested the shaking conditions for a 50 mm orbital diameter shaker to be in the range of 120-150 rpm for improved biofilm growth.

### Biofilm formation assessment by two phenotypic assays

**Fig.2:**
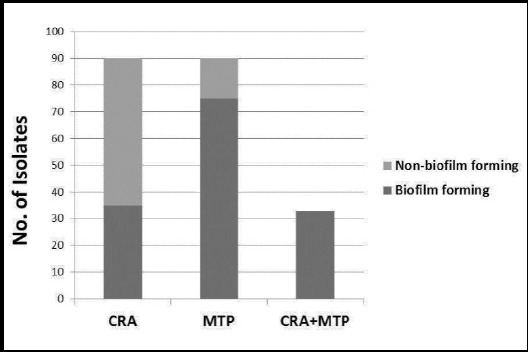
Biofilm production in *S. aureus* as assessed by Microtiter plate (MTP) and Congo red agr (CRA) methods and their correlation.

Biofilm production was accessed by two phenotypic methods: CRA (qualitative) and MTP (quantitative). Using CRA, 35 (43%) strains were rendered biofilm producers as they formed black colonies while 46 (57%) were non-biofilm producers as they displayed red colonies. In MTP, biofilm formation was detected in 75 (92%) isolates. Based on biofilm intensity we observed that 68 (91%) were moderate producers, 7 (9.3%) were strong producers, while 7 (9.3%) were non biofilm producers. When observed for correlation between two methods, it was found in 33 (30.2%) isolates (Fig.2).

### Association between icaAD genes, phenotypic assays and agr Specific Groups

**Fig.3:**
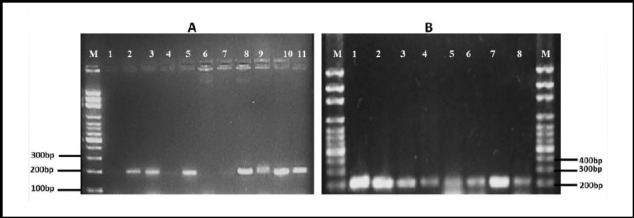
PCR amplification of extracted DNA from S. aureus with icaA icaD primers. M is 100bp Maker (A) Lanes 2-3, 5, and 8-11 demonstrate PCR products of 198bp for icaA gene. (B) Lanes 1 – 8 demonstrate PCR products of 188bp for icaD gene.

The *icaAD* genes were detected in 42 isolates (Fig.3). When correlated the phenotypic biofilm production with *icaAD* gene presence, of 42 *icaAD* gene positive isolates, 26 were positive by CRA and 40 were positive by MTP. When compared the result of MTP with *agr* genes detection, out of 65(80%) *agr* positive isolates, 60 isolates were biofilm producers among which 58 (89%) were moderate or weak biofilm producers, 2 (3%) were strong biofilm producers while 5 (8%) were non-biofilm forming. Out of 16(20%) *agr* negative isolates, biofilm production was detected in 14 isolates where 5 (31%) were strong biofilm forming, 8 (50%) were moderate biofilm forming while in 3 (19%) isolates no biofilm formation was observed.

## DISCUSSION

In our study of 81 clinical *S. aureus* isolates, biofilm production was detected in 75 (92%) strains with different intensities by MTP method. Previous studies have indicated that there is only small portion of *S*. *aureus* clinical isolates that form biofilm while few studies reported that all *S. aureus* strains tested were positive for biofilm formation.^2^,^18^ One study demonstrated that 78% of *S. aureus* were biofilm positive^19^, while another showed that 83.3% of *S. aureus* were biofilm producers.^8^ In an earlier study, biofilm-positive phenotype was exhibited by 57.8% of Staphylococcal clinical isolates and high and moderate biofilm formation was displayed by 14.47% and 39.4% strains, respectively while weak or no biofilm was detected in 46% isolates.^7^ In this study, using CRA method, 43% *Staphylococcus aureus* isolates were regarded as biofilm positive which is in agreement with other studies.^20^,^21^ The correlation between MTP and CRA was observed in 30% isolates. One study has shown low correspondence between both methods where correlation between both methods was only in 5.2% *S. aureus* isolates.^7^ In comparison, an earlier study showed better correlation where *S. aureus* positive by CRA method were also positive by MTP assay.^22^ CRA method is taken as an alternative method for screening biofilm forming *S. aureus* isolates because it is less time consuming, and easy to perform.^2^ However, our findings agree with another study that for detection of biofilm formation by *S. aureus* CRA alone should not be relied upon.^7^

Several studies have shown that biofilm associated infections in *Staphylococcus aureus* are due to presence of both *icaA* and *icaD* genes.^5^,^8^,^20^ We detected *icaA* and *icaD* genes in 42 isolates which is in agreement with studies reported in the literature.^18^ In one study *icaA* and *icaD* gene was present in 42.2% *Staphylococcus aureus* isolates.^20^ *S. aureus* isolates screened in prosthetic joint and wound infections were positive for *ica* gene.^3^,^18^ Another study demonstrated that out of 65 *S. aureus* isolates, 64 were positive for *icaA* and *icaD* genes while only one strain containing *ica* gene was found to be biofilm negative by CRA method.^20^ Similarly 54% and 52% *icaA* and *icaD* positive strains were also biofilm forming by CRA method.^3^ While comparing result of *icaAD* gene detection with MTP assay for biofilm production, 40 isolates positive for *icaAD* genes were also positive by MTP method which is in agreement with previous report.^8^ It was reported that 83.3% of *ica*-positive *Staphylococcus* isolates produced biofilm by MTP assay.^22^ Our findings suggest that PIA independent biofilm formation takes place in *S. aureus* which agrees with another report.^23^ *icaADBC* expression and PIA synthesis takes place in later stages of infection even though when microorganisms have organized in a biofilm which means that an alternative mechanism exists. A study has demonstrated that one coagulase negative *Staphylococcus* strongly adherent by MTP assay was *ica* negative.^6^ These results can be explained by the observation there are other genes such as accumulation associated protein (*aap*) and Bap homolog protein (*bhp*) that are also involved in biofilm formation.^24^ These genes were found to induce biofilm formation in PIA independent manner however, another group has reported two biofilm forming strains having neither *ica* genes nor *aap* or *bhp* genes suggesting novel mechanism of biofilm formation in these strains.^25^

Inter cellular signaling in bacteria is described by quorum sensing which also affects biofilm formation. In this study, the moderate/weak biofilm formation was observed in both *agr* negative and positive strains which indicate that *agr* has a mixed role in biofilm formation and perhaps the intensity of biofilm formation depends on the extent of expression of *agr* genes. We found that out of 65 *agr* positive strains, only 3% were strong biofilm producers, while in 16 *agr* negative strains 31% were strong biofilm producers which show that low/negative *agr* enhances the biofilm formation. This is also in accordance with the report where 78% *agr* negative while only 6% *agr* positive strains made biofilm.^12^ This suggests that to treat persistent or device related *S. aureus* infections the quorum sensing can be a target and the strategies should be to enhance the *agr* system. Furthermore, biofilm formation *in vitro* does not correlate fully with the presence of *icaAD* gene, and *ica* independent biofilm formation highlights importance of further genetic investigations.

### Author`s Contribution

**TS:** Performed the experiments and prepared draft of paper.

**MS:** Designed and performed computational simulations.

**AAK:** Performed experiments, contributed in formatting the manuscript.

**RZ:** Conception and design of research, analysis and interpretation of the data, approval of final version.
